# Male Fertility Genes in Bread Wheat (*Triticum aestivum* L.) and Their Utilization for Hybrid Seed Production

**DOI:** 10.3390/ijms22158157

**Published:** 2021-07-29

**Authors:** Manjit Singh, Marc C. Albertsen, A. Mark Cigan

**Affiliations:** 1 Corteva Agriscience, 7250 NW 62ND Avenue, P.O. Box 552, Johnston, IA 50131-0552, USA; marc.albertsen@corteva.com; 2 Genus plc, 1525 River Road, DeForest, WI 53532, USA; mark.cigan@genusplc.com

**Keywords:** wheat, hybrid wheat, male sterility, pollen development

## Abstract

Hybrid varieties can provide the boost needed to increase stagnant wheat yields through heterosis. The lack of an efficient hybridization system, which can lower the cost of goods of hybrid seed production, has been a major impediment to commercialization of hybrid wheat varieties. In this review, we discuss the progress made in characterization of nuclear genetic male sterility (NGMS) in wheat and its advantages over two widely referenced hybridization systems, i.e., chemical hybridizing agents (CHAs) and cytoplasmic male sterility (CMS). We have characterized four wheat genes, i.e., *Ms1*, *Ms5*, *TaMs26* and *TaMs45*, that sporophytically contribute to male fertility and yield recessive male sterility when mutated. While *Ms1* and *Ms5* are Triticeae specific genes, analysis of *TaMs26* and *TaMs45* demonstrated conservation of function across plant species. The main features of each of these genes is discussed with respect to the functional contribution of three sub-genomes and requirements for complementation of their respective mutants. Three seed production systems based on three genes, *MS1*, *TaMS26* and *TaMS45*, were developed and a proof of concept was demonstrated for each system. The *Tams26* and *ms1* mutants were maintained through a TDNA cassette in a Seed Production Technology-like system, whereas *Tams45* male sterility was maintained through creation of a telosome addition line. These genes represent different options for hybridization systems utilizing NGMS in wheat, which can potentially be utilized for commercial-scale hybrid seed production.

## 1. Introduction

Wheat is one of the most important and widely grown crops in the world. It is the third largest produced cereal crop, providing 20% of the world’s calories [1]. With the predicted growth in world population to over nine billion by 2050, the Food and Agriculture Organization (FAO) set a target of 60% increased food production by 2050 [2]. A continued improvement in the yield of major food crops, including wheat, is required to meet this target [3]. Breeding for hybrid varieties to exploit hybrid vigor is one way to increase stagnant yields, particularly in wheat [4,5]. Heterosis has the potential to increase yield up to 20% in wheat, highlighting the prospects for hybrid varieties [4,5,6]. Yield stability of hybrids is another major advantage of cultivating hybrid wheat. Hybrids have demonstrated superior performance compared with traditional varieties under biotic and abiotic stress conditions, thus providing opportunity to expand wheat cultivation under marginal growing conditions [7,8].

The major hindrance to large-scale commercialization of hybrid varieties in wheat is the lack of a hybridization system to economically produce hybrid seed. Wheat is an autogamous species with a perfect flower, thus excluding the option of mechanical emasculation techniques such as detasseling in maize. A biological or a chemical hybridization system, therefore, is a prerequisite for hybrid seed production in wheat. Importantly, such a system should be reliable and efficient to lower the cost of goods to produce hybrid seed. Thus far two main methods have been deployed on a small-scale in wheat to produce hybrid seed: CHAs and CMS [9,10]. Each of these systems has limitations that make large-scale deployment for commercial hybrid seed production challenging [9,10,11,12,13].

In this review we focus on a third type of male sterility system in wheat, NGMS, and its utilization for hybrid seed production in wheat. NGMS-based hybridization systems can offer several advantages over CHAs and the CMS system because of simpler genetics of male sterility and fertility restoration [2,14]. Over the past several decades many male sterile mutants have been identified in wheat and male sterility genes cloned, including four genes that have been characterized by our group at Corteva^TM^ Agriscience in partnership with external collaborators. Based on these and other male fertility genes, several NGMS hybridization systems have been proposed and are discussed in this review.

## 2. Sporophytic Genes Involved in Anther and Pollen Development in Wheat

Flowering plants have developed specialized structures to produce male and female gametes to accomplish sexual reproduction. Successful production of male gametes relies on proper formation of male reproductive organs. Pollen grains or microgametophytes are formed in the anthers, the male reproductive organ, and deliver male gametes to organs bearing female gametes (reviewed in [15]). Pollen grains are surrounded by protective pollen walls, intine and exine to enable survival of pollen in diverse environmental conditions. The intine is composed of cellulosic material, whereas sporopollenin is the major component of the exine (reviewed in [16]). Sporopollenin fortifies the exine through formation of a skeletal structure and a durable covering. The components of the exine are synthesized by the surrounding tapetum and deposited on the surface of developing microspores [17].

NGMS arises due to mutations in the genes involved in anther and sporophytic stages of pollen development, including sporopollenin biosynthesis pathway. Spontaneous male-sterile mutants have been reported in more than 175 species of angiosperms [18]. Of the mutants that have been characterized, more than 60% are attributed to a single recessive gene [18]. In addition to the spontaneous mutants, several sporophytic male fertility genes have been identified through mutant screens (reviewed in [19,20,21]) (Table 1).

### 2.1. Male Sterile Mutants in Wheat Identified through Forward Genetics

In hexaploid bread wheat, the three genomes A, B and D exhibit extensive functional redundancy, with all three homeologs contributing towards many traits. Therefore, a triple knockout of all three genomes is often required to uncover a mutant phenotype [32,33,47]. Due to this functional redundancy, few male sterile mutants have been identified in wheat compared with single genome diploid species such as maize, rice and barley. Nevertheless, a few male sterile mutants have been identified through forward genetics, either as spontaneous mutations or through mutagenesis (http://www.shigen.nig.ac.jp/wheat/komugi/genes/symbolClassList.jsp) (accessed on 6 May 2021). Genetic studies and subsequent cloning of genes for many of these mutants has revealed these as either dominant mutations or mutations in genes with a predominant functional homeolog (Table 1). *ms1* and *ms5* are two such recessive mutants where one genome predominantly contributes to gene function [25,27,31]. *ms1a* mutant was the first male sterile mutant reported in wheat [48]. Subsequently, various forward genetic screens have identified seven *ms1* mutant alleles (*ms1a-g*). Dominant male sterility can arise due to mutations in a single homeolog; therefore, more dominant mutants, such as *Ms2*, *Ms3* and *Ms4*, have been identified as compared with recessive mutants. Of the dominant mutants, *MS2* encodes an orphan protein that is specifically activated in the *Ms2* mutants through the insertion of a retro-element in the promoter of the gene [28]. Tang et al. [49] utilized CRISPR/Cas9 to edit *Ms2* to restore male fertility to *Ms2* mutant. The genes for the *Ms3* and *Ms4* have not been identified yet.

### 2.2. Male Sterile Mutants in Wheat Revealed through Reverse Genetics

The role of an increasing number of wheat genes in male fertility is now being revealed through reverse genetics (Table 1). The availability of more efficient gene editing technologies, such as CRISPR/Cas, has accelerated the functional analysis of wheat genes, as it is possible to obtain concurrent mutations in three homeologs [33]. It is not surprising that all the genes investigated through reverse genetics exhibit functional redundancy of the three homeologs. Utilizing the new gene editing tools, wheat homologs of the maize *MS26* and *MS45* genes were characterized for their role in male fertility [32,33,50]. For both these genes, knockouts of all three homeologs are required for complete loss of function, which resulted in male sterility. Similarly, it has been observed that for *TaNP1*, the wheat homolog of the rice *OsNP1* and maize *ZmIPE1* genes encoding a putative glucose-methanol-choline oxidoreductase, knockouts in all three homeologs are required for male sterility [34]. Milner et al. [36] identified two male fertility genes in wheat, *CALS5*- and *RPG1-like*, through gene expression analysis. Functional analysis of these genes through CRISPR/Cas showed that knockouts in all three homeologs are required to obtain male sterile plants [36]. Similarly, triple knockouts of all the three homeologs for *TaTDF*, the wheat homolog of the Arabidopsis *TDF1 (Tapetal Development and Function1*)*,* are required to completely abolish function and achieve male sterility [35].

### 2.3. Analysis of Male Fertility Genes Ms1, Ms5, TaMs26 and TaMs45

**Figure 1:**
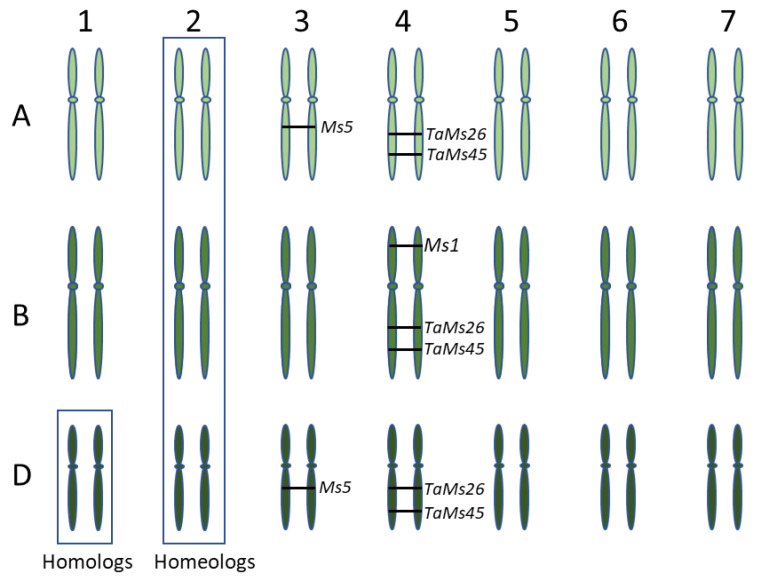
Diagrammatic representation of the three wheat sub-genomes illustrating homologs and homeologs. The numbers at the top indicate the homeologous chromosome group, while alphabets on the left indicate the three sub-genomes. Position of functional homeologs of four wheat fertility genes, *MS1*, *MS5*, *TaMS26* and *TaMS45* is indicated.

To understand the genetic basis of male fertility in wheat, we analyzed four genes, *MS1*, *MS5*, *TaMS26* and *TaMS45*, that sporophytically contribute to male fertility in wheat (Table 2). The putative function of these genes suggests a role in tapetum function, and thus pollen development, and their loss of function causes defective tapetum and pollen development. *MS1* and *MS5* genes were identified through positional cloning of *ms1* and *ms5* mutants. *TaMS26* and *TaMS45* are homologs of the maize male fertility genes *MS26* and *MS45*. *MS1*, *TaMS26* and *TaMS45* are located on the group 4 chromosomes, while *MS5* is located on group 3 chromosomes (Figure 1). The number of functional homeologs varied from one to three for each the four wheat fertility genes (Figure 1).

Genetic studies with several *ms1* alleles showed monogenic recessive segregation of male sterility attributed to chromosome 4 BS [2]. *MS1* was cloned through positional cloning and was determined to encode a glycosylphosphatidylinositol-anchored lipid transfer protein (LTP), necessary for pollen exine development [27].

Gene expression analysis revealed that only the B-genome-derived *TaMS1* homeolog is expressed during microspore development. Microscopic analysis and metabolomic profiling of anthers from *ms1* mutants suggested the requirement of *TaMS1* for either biosynthesis or transport of sporopollenin, and hence pollen exine formation. It was also demonstrated that an exogenous copy of *TaMS1* completely restored fertility to the ms1d mutant, confirming *TaMS1* as the causal gene for *ms1* mutation [27]. Similarly, *TaMS5-A*, the gene responsible for *ms5* phenotype, was also cloned using positional cloning, and like *TaMS1*, encodes a glycosylphosphatidylinositol-anchored LTP required for pollen exine development [31]. Phylogenetic analysis showed that *MS1* and *MS5* proteins are distinct from the other members of the wheat LTP super-family [51]. Sequence homology and gene expression pattern of *MS5* were also unique to Triticeae and highly distinct from the homologs outside the Triticeae family [31]. Like *ms1d*, *ms5* was also defective in exine formation of the microspores, although an irregular nexine structure was unique to the *ms5* mutants. Like *TaMS1*, an extraneous copy of *TaMS5-A* homeolog was sufficient for complementation of *ms5* mutants to restore male fertility [31].

The maize *MALE STERILE26* (*MS26*) gene encodes a cytochrome P450 mono-oxygenase enzyme, CYP704B1 [52]. Subfamily CYP704 of the cytochrome P450s has an essential role in male fertility through hydroxylation of the fatty acid constituents of predicted sporopollenin precursors [21]. *ms26* mutants are male sterile due to defective tapetum and microspores that lack sporopollenin deposition on the exine [52,53]. Gene expression analysis in wheat revealed that all three *TaMS26* homeologs are expressed in the anthers from tetrad to early uninucleate microspore stages at comparable levels [32]. Mutations in the A, B and D homeologs of the putative *MS26/CYP704B* wheat gene were obtained utilizing a meganuclease [50]. Similar to the maize *ms26* mutants, triple homozygous wheat mutants were male sterile due to defective pollen and anther development [32]. Expression and functional analysis confirmed the contribution of all three homeologs of *TaMS26* towards male fertility in wheat. Interestingly, a single heterologous *MS26/CYP704B* gene was unable to restore fertility to a triple homozygous mutant background, but two heterologous genes completely restored male fertility [32].

The maize *MALE STERILE45* (*MS45*) gene encodes a strictosidine synthase-like enzyme required for male fertility [54,55]. Analysis of CRISPR-Cas derived *TaMS45* mutations demonstrated that all three homeologs contribute to male fertility and that triple homozygous mutants are required to abort pollen development and achieve male sterility [33]. Further, it was demonstrated that a single wild-type copy of the *MS45* gene from rice or maize was able to restore fertility to the male sterile wheat mutants, suggesting a conservation of function between these species [33]. This conservation in expression and function was also utilized to complement the *TaMS45* promoter inverted repeat (pIR)-based dominant male sterility through the expression of maize *MS45* with maize or rice *MS45* promoters [56].

### 2.4. Ms1, Ms5, TaMs26 and TaMs45 Exhibit Different Levels of Functional Redundancy

Bread wheat is an allohexaploid (2*n* = 6× = 42) consisting of three ancestral genomes (AABBDD), formed following two hybridization events between three progenitor species [57,58,59]. As a consequence of polyploidization and sub-functionalization, asymmetrical genetic contribution of the three subgenomes is an important characteristic in wheat [1,60,61]. Interestingly, the four male fertility genes we analyzed represented a complete spectrum of dosage of the three sub-genomes towards male fertility (Table 2). While *MS1* and *MS5* have one and two functional homeologs, respectively, *TaMS26* and *TaMS45* both have three functional homologs. Similarly, these genes also displayed a difference in the minimum number of wild-type alleles required for male fertility and the number of transformed wild-type genes required for complementation of their respective mutants (Table 2).

*ms1* and *ms5* both show monogenic inheritance; however, the underlying basis for this inheritance is different for both these genes, with *ms5* presenting a more complex control of male fertility. In the case of *MS1*, sub-functionalization occurred due to inactivation of the A and D genome homeologs, with B genome being the only functional homeolog [27,62]. DNA methylation analysis of *MS1* promoter of A, B and D homeologs, along with orthologs from allotetraploid and diploid wheat species, showed that the A and D homeologs are epigenetically silenced with only the B homeolog expressed in the anthers [62]. Therefore, a knockout of the B-genome homeolog is sufficient to abolish *MS1* function and result in male sterility. However, variable penetrance of male sterility has been reported in various *ms1* mutants [27]. This variation could be due to activation of the A- and D-genome homeologs, although this needs to be further investigated. For *MS5*, the predominant functional homeolog is *TaMS5-A* [31]. *TaMS5-D* exhibits has two allelic forms, one of which is non-functional, while the second form, although functional, displays incomplete dominance, suggesting reduced functionality [31]. Consistent with observed differences in function, the transcript abundance in developing anthers is lower for *TaMS5-D* than *TaMS5-A*. The non-functional form confers monogenic inheritance of male sterility, while the second form displays a two-gene inheritance [31]. Therefore, depending upon the *TaMS5-D* allele present, knockout of either one or two *TaMS5* homeologs is required for achieving male sterility. For the *TaMS5-B* homeolog, two different non-functional alleles were observed in the 178 diverse hexaploid and tetraploid wheats analyzed, including several landraces and *Triticum dicoccoides* [31]. The prevalence of the non-functional *TaMS5-B* alleles in diverse hexaploid and tetraploid wheats suggests an early elimination of function of this homeolog during evolution. Thus, *TaMS5* homeologs appear to have undergone two subsequent steps of sub-functionalization: an ancestral inactivation of *TaMS5-B* and a more recent reduced functionality of *TaMS5-D*.

**Figure 2:**
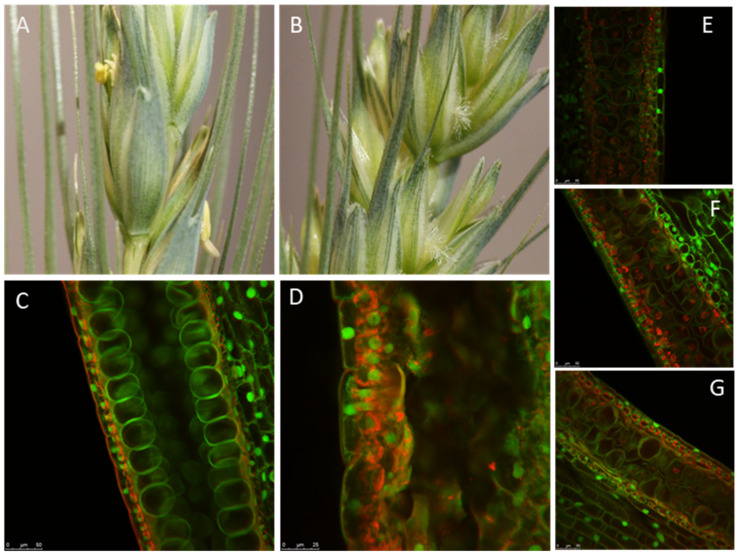
Functional analysis of *TaMS26* mutant wheat plants. Spikes from (**A**) wild-type and (**B**) *Tams26-abd* mutant plants. Microspores at late vacuolate stage from (**C**) wild-type *TaMS26*-*ABD*; (**D**) triple-recessive *Tams26-abd*. Double homozygous-single heterozygous: (**E**) *Tams26-Aabd*, (**F**) *Tams26-aBbd* and (**G**) *Tams26-abDd* plants. (**E**), (**F**) and (**G**) illustrate the differences in pollen morphology of double homozygous-single heterozygous mutants that are heterozygous for A-, B- and D-genomes, respectively. Scale bars = 25 μm. (Adapted from Singh et al. [32].)

Both *TaMS26* and *TaMS45* genes have three functional homeologs, as demonstrated by the requirement of triple knockouts to achieve a complete loss of function. However, for *TaMS26*, an in-depth characterization revealed differences in functional contribution of A, B and D homeologs [32]. In addition to the triple mutants, it was observed that double homozygous-single heterozygous mutants also exhibited male sterility but with varying levels of residual fertility (Figure 2). The fertility of these triple mutants was dependent upon the homeolog contributing the wild-type allele. This residual fertility was the highest in plants heterozygous for D genome and lowest in plants heterozygous for B genome (Figure 2). Genome A heterozygotes were intermediate for residual male fertility. Therefore, the B homeolog contributes the least towards male fertility, whereas the D homeolog contributes the most [32]. Thus, when a single wild-type allele of each homeolog was analyzed against five mutant alleles, differences appear in contribution of each genome. This also suggested that two wild-type alleles out of six *TaMS26* alleles are required for complete male fertility, with the two alleles contributed by one genome or a combination of any two genomes [32]. Compared with *TaMS26*, only one wild-type allele is required for complete fertility *TaMS45* [33]. This was evident from the analysis of fertility in double homozygous-single heterozygous *TaMS45* mutant plants and from complementation analysis of triple mutants. This difference in the function of *TaMS45* and *TaMS26* highlights the functional differences between the homeologs of these genes, which could be ascribed to transcriptional, translational or enzymatic differences. Thus, *MS1*, *MS5*, *TaMS26* and *TaMS45* represent different aspects of polyploid biology, which is important to understanding how to create hybridization control systems.

### 2.5. Temperature and Photoperiod Influenced Male Fertility

In addition to the non-conditional genetic mutants described above, two types of environmentally influenced nuclear genes have also been reported in wheat that affect male fertility (Table 1). Thermo-sensitive genetic male sterility (TGMS) is controlled by genes that are influenced by temperature, while photoperiod- and temperature-sensitive genetic male sterility (PTGMS) is influenced both by temperature and photoperiod. TGMS and PTGMS variants have been mainly identified by wheat researchers in China. Four mutants have been reported thus far that can be classified in the TGMS category. YanZhan 4110S is one such line where male fertility is sensitive to temperature during late uninucleate stage of microspore development, with complete male sterility at temperature above 20 °C [38]. Two genes, *TaMUT11* and *TaSF3*, have been reported to be associated with TGMS in YanZhan 4110S [63]. BNY-S is another TGMS line identified as a spontaneous mutant of the fertile wheat line BNY-F [39]. BNY-S exhibits male sterility at temperatures less than 10 °C during spikelet differentiation stage but is male fertile at temperatures higher than 10 °C. Genetic analysis indicated that sterility in BNY-S was controlled by a single recessive gene, wtms1 [39]. TGMS line tmsBS20T has also been used for hybrid wheat breeding in northern China [41]. Male sterility in tmsBS20T segregates as a single gene that was mapped to chromosome 2BL. BNS, another TGMS line identified in wheat, exhibits male sterility in the temperature range of 7.4 °C to 11.4 °C but is male fertile at temperature higher than 11.4 °C. [40,64].

In addition to TGMS mutants, several PTGMS lines have also been identified in wheat. BS366 is one such line that is the female parent of several hybrid varieties developed through two-line breeding system [44,65]. BS366 exhibits male sterility under a sterile condition of 10 °C with 12–14 h daytime during pollen development but fertility at 20 °C with 12–14 h daytime [44]. C49S, another PTGMS line, is also being utilized in two-line hybrid wheat development in China [45]. The critical temperature to induce complete male sterility is less than 13.5 °C, with near normal male fertility at 15 °C or higher. Two improved PTGMS lines, K78S and C412S, were subsequently derived from C49S [66,67]. BS210 is a PTGMS line that is male sterile between 10–12 °C and a photoperiod of 10–12.5 h during the critical stages of anther development. It exhibits up to 70% fertility at temperatures of 8–10 °C and 14 h photoperiod [43]. Male sterility in Xinong 291S (XN291S), another PTGMS line, is controlled by one or two recessive major genes [46].

## 3. Hybridization Systems for Hybrid Seed Production in Wheat

In the past six decades of hybrid wheat research, two main systems for hybridization have been the focus of wheat breeders, i.e., CHAs and CMS. However, NGMS-based hybridization systems can provide certain advantages over these two systems, which makes NGMS systems very attractive. The unique characteristics and the major challenges for each of these systems are discussed below.

### 3.1. CHA-Based Hybridization System

**Figure 3:**
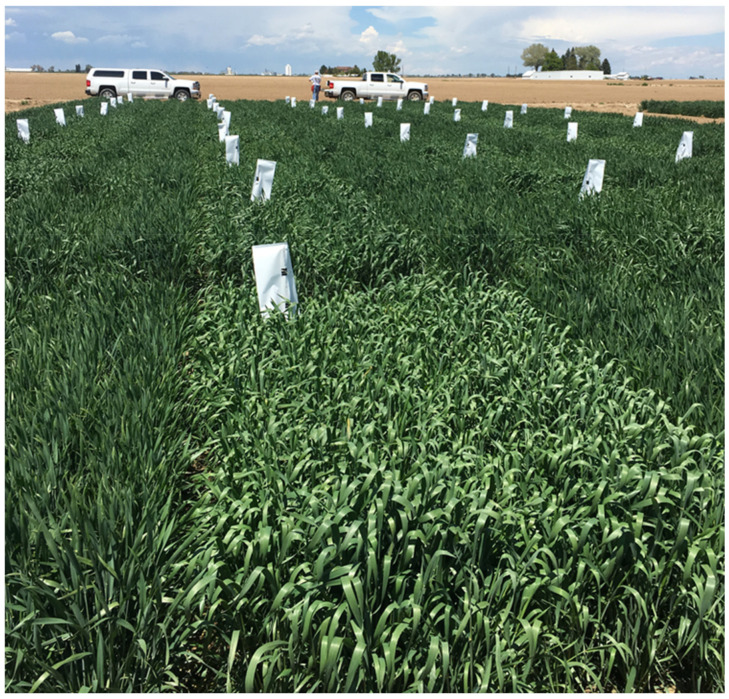
Utilization of CHAs for hybrid seed production in wheat. The rows with white bags represent the male-sterile female parent treated with CHA. The unbagged rows represent the male-fertile male parent. (Photograph courtesy of Bill Curran, Corteva^TM^ Agriscience).

Chemical hybridizing agents are a class of chemical compounds, also known as gametocides, that can selectively induce male sterility without affecting female fertility (Figure 3). The major advantage of CHAs for hybrid seed production is the elimination of the need of any genetic manipulation for creating male sterile lines, which can effectively reduce the cost of goods to produce hybrid seed. The research for utilizing CHAs in wheat started with the first generation of CHAs, which included many commonly used growth hormones. However, these chemicals were highly variable in efficacy and exhibited phytotoxic effects [2]. This led to the discovery, mainly by the chemical industry, of much improved second generation CHAs [68]. Gametocide Hybrex (active substance RH 007 CHA) was invented by Rohm & Haas, compound SD 84811 was invented by Shell/Nickerson, Genesis® (active substance - MON 21200, Clofencet) was discovered by Monsanto, and Croisor® (active substance—Sintofen) was discovered by DuPont. These second generation CHAs, which are effective across a broad range of genotypes and have reduced phytotoxicity, have been reasonably utilized for hybrid seed production in wheat. This is evident from registration of seventy-four hybrid wheat varieties that were produced through the use of CHAs from 1996 to 2016 [68].

Still, the use of CHAs is limited due to a number of factors that restrict their large-scale adoption. The narrow window of CHA application, which can be severely affected by prevailing environmental conditions, is the major limitation to utilizing CHAs. This can make the application of CHAs challenging and therefore risky for seed production. Emergence of late tillers that can escape application of CHAs adds to that risk of contamination with self-pollinated seed. Phytotoxicity associated with CHAs also necessitates dosage optimization for diverse inbreds. Another key drawback of a CHA-based system is the inability to utilize blend planting of male and female parents for hybrid seed production (Figure 3). With the current available technologies, strip planting of male and female lines is the only option for a CHA-based hybridization system, as opposed to male and female blend planting proposed for genetic hybridization systems to maximize cross pollination [69,70]. The restriction of using strip planting necessitates the development of super males with exceptional male characteristics, to get optimal hybrid seed production. Consequently, current hybrid wheat breeding programs utilizing CHAs rely on a few male inbreds [68]. However, with the advent of advanced technologies for pollen preservation and application, it may be possible to increase cross pollination even for CHA-based hybridization systems [70]. Nevertheless, even with the most effective CHAs, any variation due to plant growth or application makes utilization of CHAs for hybrid seed production challenging.

### 3.2. Genetic Hybridization Systems

The genetic hybridization systems rely on genetic factors for the induction of male sterility, without the need for any external manipulation or application. Two main types of genetic male sterility systems have been utilized for creating hybridization systems: CMS and NGMS.

#### 3.2.1. CMS Hybridization System

**Figure 4:**
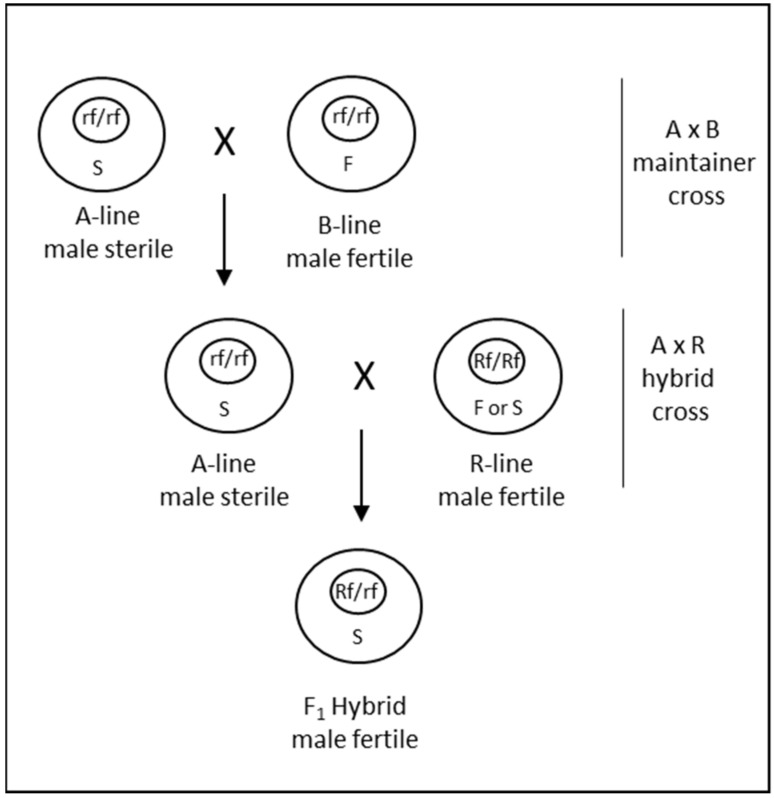
Diagrammatic representation of the CMS system for hybrid seed production in wheat. The bigger outer and the smaller internal circles represent the cytoplasm and the nucleus, respectively. S and F denote the sterile and fertile cytoplasms; *Rf* and *rf* denote the fertility restoring and non-restoring alleles of the restorer gene, respectively. A, B and R lines represent the male-sterile female parent, the male-fertile female isogenic maintainer and the restorer male parent, respectively.

Cytoplasmic male sterility occurs due to the interaction of nuclear and cytoplasmic genetic factors [71]. This generally occurs when cytoplasm from a distantly related species is combined with the nucleus of another species, resulting in an alloplasmic line. The male sterility can be reversed with restorer of fertility (*Rf*) genes, which in many instances are derived from the species that contributed the cytoplasm. In addition to the male sterile A-line and the male fertile R-line, an additional B-line is also required for the maintenance of male sterility through A- × B-line cross (Figure 4). Thus, a CMS system is a 3-line system that requires cross-pollination at two steps to produce hybrid seed. Since its discovery in wheat in 1951 [72], CMS has been extensively researched with the aim of utilizing it for commercial seed production. The cytoplasms of various wheat-related species have been combined with the nucleus of *T. aestivum* to investigate CMS. Tsunewaki et al. [73] analyzed 46 cytoplasms transferred to wheat from *Triticum* and *Aegilops* species, of which 31 could induce partial to complete male sterility. Other studies have also demonstrated the ability of cytoplasm from many species to induce CMS when combined with the *T. aestivum* nucleus [74]. Due to the stability of male sterility, predominance of recessive alleles for restoration and identification of corresponding dominant restoration factors, CMS induced by *T. timopheevii* Zhuk. has been primarily considered for commercial hybrid seed production in wheat [69]. Several hybrid varieties were commercialized from the late 1970s to the 1990s utilizing a *timopheevii* CMS system [74].

However, there are certain aspects of CMS systems, including *timopheevii* CMS, that render these systems more complex for hybrid seed production in wheat. First, various studies have revealed that combinations of two or three and perhaps more *Rf* genes are needed for complete fertility restoration of CMS in wheat [75,76,77]. To add to that complexity, epistatic interactions among *Rfs* and between *Rfs* and modifier loci have also been highlighted by several studies [78]. Consequently, it is required to stack multiple *Rf* genes in a genotype-dependent combination to achieve complete fertility of F_1_ hybrids. In the past, stacking and breeding of restorer genes has proved challenging, although with contemporary genomics and molecular breeding techniques it is now possible to precisely track *Rf* genes for breeding [79]. Another major limitation for utilizing a CMS for hybrid seed production is the requirement for A × B crossing for female parent seed increase (Figure 4). The strip plots used for A × B female-increase limit the efficiency of female inbred multiplication. For such a maintainer crossing, it is necessary that the B line should also have good male characteristics. This adds another layer of complexity to breeding for female parents. Several undesirable pleiotropic effects that are environment-dependent have also been reported for *timopheevii* cytoplasm, including shriveled F_1_ seed [13]. All these factors can lead to the increased cost of goods for hybrid seed production, which is likely the reason that currently no hybrid wheat varieties that were produced utilizing a CMS system are commercially available.

#### 3.2.2. Hybridization Systems Based on NGMS

An alternative to the utilization of CHAs or CMS are the systems based on NGMS (Table 3). There are two major advantages of hybridization systems based on the recessive NGMS. First, no genetic manipulation, trait introgression or breeding is required of the male inbreds for fertility restoration. Since most of the mutants or variants of the nuclear genes are recessive in nature, restoration of fertility can be achieved in the heterozygous F_1_ hybrid. This is also true in cases where triple homeolog mutants are required to generate male sterile lines. Therefore, in terms of restoration of male sterility, NGMS is much simpler compared with CMS. Dominant or semi-dominant NGMS systems, however, require a more complex strategy for restoration of male fertility. The second advantage of NGMS based hybridization systems is the propagation or maintenance of the male-sterile female lines through self-pollination of the maintainer lines. As mentioned previously, maintenance of male sterile plants in an A × B crossing scheme for CMS adds complexity to the hybridization system and increases the cost of goods. Thus NGMS, including both conditional and non-conditional, can offer an advantage for a hybridization system in wheat.

##### TGMS- and PTGMS-Based Hybridization Systems

While several TGMS and PTGMS lines have been characterized in wheat (Table 1), few are being utilized for hybrid seed production [70]. BS366 is an important PTGMS line for a hybrid wheat breeding program in China and is the female parent of Jingmai-series hybrid wheat varieties that include Jingmai 7 (JM7), Jingmai 8 (JM8) and Jingmai 9 (JM9) [44,65]. A major concern for large-scale implementation of TGMS and PTGMS is the effect of sudden changes in temperature and/or photoperiod on male sterility and fertility of the female lines. Any leakiness of sterility due to temperature or photoperiod fluctuations will result in contamination of hybrid seed with female parent seed. Similarly, any variation from the required conditions can reduce the seed set of female inbreds during maintenance, resulting in supply chain issues. Spreading seed production over a number of locations can alleviate the risks associated with temperature or photoperiod variation. However, the number of such locations may be limited due the narrow range of climatic zones where the hybrid seed production and female maintenance can occur. Penetrance of sterility in TGMS/PTGMS variants may also be a concern for their utilization for hybrid seed production. It is reported that under sterile conditions PTGMS lines BS366 and BS210 exhibit 95–100% male sterility, suggesting up to 5% fertility under sterile conditions [43,44], which can impact purity of hybrid seed. Another issue, which appears to be wheat specific, is the manifestation of TGMS and PTGMS, in general, at lower temperature, with fertility occurring at higher temperatures. This contrasts with the classical TGMS mutants in other crops, where TGMS manifests at higher temperatures [80]. Observations similar to wheat have also been reported in barley [81], suggesting that this may be a common feature of the *Triticeae* family. This feature can be an obstacle to utilizing TGMS and PTGMS for hybrid seed production in wheat since both the spring and winter wheat flower in spring when the daily temperatures are rising. This can decrease the penetrance of male sterility and enhance reversion to fertility of female lines. Restoration of sterility in F_1_ hybrids generated through TGMS and PTGMS can also be complex [44,82]. Thus, the TGMS and PTGMS systems have several drawbacks that may limit their application for hybrid seed production in wheat.

##### Hybridization Systems Based on Non-Conditional Recessive NGMS

Following the discovery of the first nuclear male sterile mutant in wheat [48], the first NGMS hybridization system was proposed in 1972 [83]. This system, known as the XYZ, can be described as the first-generation concept for utilizing NGMS for hybrid seed production. It was based on a recessive male-sterility mutant, *ms*, that could be complemented with a homeologous chromosome derived from a wheat-related species to render it male fertile. X, Y and Z, the three lines required for maintenance for male sterile females, are all homozygous for the male sterile mutation (*ms/ms*) but carry 2, 1 and 0 doses of the alien chromosome, respectively [83]. In a hypothetical example, Driscoll considered a male sterile mutant of homeologous group 5 for wheat that could be complemented with the addition of rye chromosome 5R, which also carries a marker gene for hairy peduncle [83]. While this concept was a major step forward for utilizing NGMS for hybrid wheat seed production, the selection of *ms/ms* plants at a vegetative stage would be difficult to implement in the field. A modified XYZ was later proposed that did away with the need for the X component [84]. However, this system also required a Z × Y crossing step, similar to the A × B cross in the CMS system, for the maintenance of the male sterile female, thus making it a 3-line system (Table 3).

**Figure 5:**
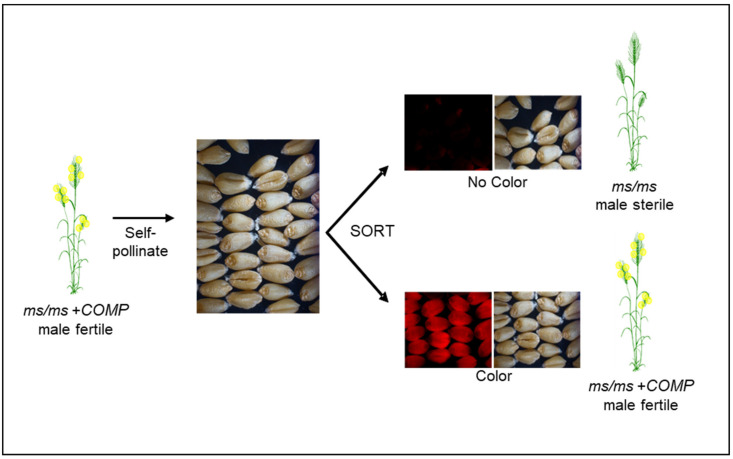
Diagrammatic representation of a NGMS system based on seed color marker for hybrid seed production in wheat. The *ms/ms* and *COMP* denote the male sterility and complementing components, respectively. The male sterility and complementation components including seed color can originate from different sources that can be combined through genetic manipulation.

Since the mid-2000s, several groups have refocused attention on the development of NGMS-based systems that can be described as second-generation concepts for utilizing NGMS. Zhou et al. [85] devised the 4E-ms system, which is a further modification the XYZ system (Table 3). The selection for *ms/ms* plants is based on seed phenotype, which predicts the genotype of the plant derived from that seed (Figure 5). In this system, the *ms* component, a mutant allele of the *MS1* gene, was combined with the complementation component, 4E chromosome from *Agropyron elongatum* ssp. Ruthenicum, essentially creating a monosomic addition line [85]. Chromosome 4E also has the *BLUE ALEURONE* (*BA*) gene, which imparts a light blue color to the seed containing the 4E monosomic. In a working system, the background is always *ms1/ms1* for the *ms* component, but the plants are fertile due to presence of the 4E chromosome. A plant derived from a light blue-colored seed, upon self-pollination, segregates for three categories of seed—red, light blue and blue carrying, 0, 1 and 2 doses of the 4E chromosome [85]. The proportion of red and blue seeds is about 65% and 35%, respectively, where the red seed represents the male sterile females for hybrid seed production, while the light blue seed serves as the female maintainer line.

Although an excellent concept, the commercial use of the 4E-ms system has not been reported. This may be due to a few inherent shortcomings of this system that relate both to the *ms* and the 4E complementation components. The first issue can be attributed to the location of *MS1* and *BA* components on the 4E chromosome. If the synteny of genes holds true between the wheat group 4 chromosomes and 4E, the *MS1* and *BA* gene are likely to be on opposite arms. It is known that the monosomics form univalent chromosomes during meiosis and tend to break at centromeres during meiosis. Therefore, it is possible that due to breakage at the centromere, *MS1* and *BA* can be unlinked, resulting in segregation and thus misclassification of red and blue seed. This can result in red seeds producing male fertile plants and male sterile plants derived from blue seeds. Several studies have also suggested variation in the penetrance of *ms1* mutations [27,90,91]. This could also lead to the presence of female inbred seed in the hybrid seed, depending on the penetrance of the *ms* component. Finally, the efficiency of such a system relies on the ability to sort various classes of seeds, i.e., red, light blue and dark blue seeds, particularly the ability to efficiently remove blue seeds disomic for 4E chromosome. Any environmental or genotypic variation in blue color will further increase the complexity of seed sorting, thus reducing the efficiency of this system.

Seed production technology (SPT) [55], initially developed for corn at Corteva^TM^ Agriscience, can mitigate the linkage issue associated with the 4E-ms system (Figure 5). In the SPT system, the complementation component and the seed marker reside on the same TDNA insertion, ensuring absolute linkage of these components. The pollen-specific expression of alpha-amylase gene, another component of the TDNA, prevents the transmission of TDNA construct through pollen. Therefore, the issue of homozygous TDNA, equivalent to the disomics in 4E-ms system, is not encountered. When a *ms/ms* plant hemizygous for the TDNA is self-pollinated, all the seeds have a *ms/ms* genotype, half of which carry the TDNA, with the other half being devoid of TDNA. Each class of these seeds can be sorted based on the seed marker. *ms/ms* seeds without TDNA will produce a male-sterile female parent to cross with a male parent for hybrid seed production (Figure 5). An important aspect of this system is that the female parent, and thus the hybrid seed and commercial grain, is devoid of TDNA and is therefore SPT transgene-free.

Based on the maize SPT concept, we have assembled wheat hybridization systems with two male sterility mutants, *ms1*, and *Tams26* (Table 3). Additionally, components for two more systems based on *ms5* and *Tams45* mutants have been determined. The first such hybridization system was reported for the *TaMS26* gene [32]. Homozygous *Tams26* mutants formed the *ms* component, whereas the complementing piece was provided by a TDNA cassette that contained a *ZmMS26*-*OsMS26* gene combination, DSRED as a seed marker and a pollen-specific alpha-amylase gene [32]. When *Tams26* plants with TDNA cassette were self-pollinated, the obtained seeds segregated approximately 50:50 for with and without a TDNA cassette (Figure 5). All *Tams26* plants that carried a TDNA cassette were male fertile and set seed comparable with wild-type plants; all plants without the T-DNA cassette were male sterile [32]. The TDNA carrying seeds could be sorted by a seed sorter appropriate for the seed marker. (Figure 3). A similar system was established with *ms1* as the *ms* component and a TDNA cassette containing *TaMS1*, DSRED and the pollen-specific alpha-amylase gene as the complementing piece (M. Singh, M. Albertsen, M. Cigan, unpublished results). *ms1/ms1* plants with a TDNA cassette are completely fertile and upon self-pollination produce seeds that segregate approximately 50:50 for with and without TDNA. All the plants produced from seeds without the seed marker were male sterile, while those from seeds with the marker were fertile. *ms1/ms1* seeds without the TDNA generated a male-sterile female parent to be crossed to a male parent for hybrid seed production (Figure 3). In addition to *ms1* and *Tams26* systems, the complementation component has been demonstrated for *ms5* and *Tams45* mutants [31,33]. These proof-of-concept studies showed that a SPT-like hybridization system is feasible in wheat.

Another hybridization system for wheat has been described by [86] through tapetum-specific expression of the phytotoxic barnase gene. The barnase gene was split into two components, each of which is placed at the same locus in separate plants, thus representing two alleles. Male sterility is induced when the two components or alleles of the barnase gene are brought together through crossing [86]. The major advantage of this system is that, like the systems based on recessive mutants, no restorer genes are required; therefore, genetic modification of male parent is not needed. However, the maintenance of this system requires an A x B like crossing scheme akin to a 3-line CMS system. The F_1_ hybrids still carry the transgene in a heterozygous state and therefore will be regulated as a transgenic trait.

Another hybridization system proposed for wheat is based on the chemical complementation of mutants in known biochemical pathways. Sedlacek [37] demonstrated that the inactivation of *TaSPPS*, a gene involved in the biosynthesis of sporopollenin, results in male sterility. This sterility can be reversed through application of 4-oxo-6-octadecane-pyran-2-olate at the tetrad stage of pollen development [37]. Like TGMS and PTGMS, such a system will be considered a classical 2-line hybridization system. However, there are no reports of this system being implemented at a commercial scale for hybrid seed production, with one of the hurdles to overcome being the registration of the chemical for commercial use.

Recently, a few hybridization systems have been proposed that seek to build up on the first- or second-generation hybridization concepts (Table 3). These concepts specifically aim to tighten the linkage between the complementation and the selection/sorting components through either native or gene editing techniques. Ms45-BA is a hybridization system based on the mutations in the *TaMS45* gene as the *ms* component, and the telosome for long arm of 4E (t4EL) from *Thinopyrum ponticum* as the complementation piece [87]. It was previously shown that mutations in all three homeologs of the *TaMS45* gene are required for achieving male sterility [33]. It was further shown that the *Tams45* triple mutants can be complemented with heterologous *MS45* genes from maize and rice, suggesting the potential of *MS45* genes from a diverse range of species to complement *Tams45* mutants [33]. Chromosome 4EL of *Thinopyrum ponticum* is homologous to chromosome 4L of wheat [92], where *TaMS45* is located. Because of this homology, it was anticipated that 4EL should carry a homolog of the *TaMS45* gene in addition to the *BA* gene for blue aleurone. Studies in our group demonstrated that the t4EL can both complement the *Tams45* triple mutants and impart blue color to the seed [87]. Both these components are tightly linked due to the lack of recombination between t4EL and the wheat 4L group and the low probability of chromosome breakage that occurs with monosomic addition lines. Self-pollination of Ms45-BA plants yields red and blue seed ratios similar to those described for 4E-ms [85].

In addition to the Ms45-BA system, several concepts for hybridization systems in wheat have been proposed that rely heavily on chromosome engineering. Two hybridization concepts have been proposed to further modify 4E-ms system by moving the *MS1* and *BA* components closer for a tighter linkage, either by conventional genetic techniques or utilizing gene editing technology [87,88]. Sedlacek and Horcicka [14] have proposed an updated XYZ hybridization system based on the *TIP2* gene as the *ms* component and the barley 7H chromosome as the complementation component. The proposed system utilizes the amylose profiles of the wheat *waxy* null-mutants and the 7H monosomic addition line, which has a WT waxy allele, to sort for seed [93]. The male sterile line increase is proposed by crossing to the 7H fertile maintainer in an A × B crossing scheme, similar to a 3-line system. The self-pollination of the 7H addition line for maintainer increase, however, could pose problems, as the seed with 0, 1 and 2 doses of the monosomic will have to be sorted into each class to eliminate disomic seeds. Another hybridization concept has been recently proposed that relies heavily on gene editing technology [89]. The *MFW-PV-OV* Maintainer Line concept intends to create male-fertile maintainer lines that can be propagated by self-pollination without the need for seed or plant sorting [89]. However, the male sterile lines must be crossed to male-fertile maintainer plants in an A x B-like crossing scheme similar to a 3-line system. For the wheat version of this system, it is proposed to knockout 5–6 alleles of three genes, *MFW*, *PV* and *OV*, along with intra-chromosomal deletions.

##### Hybridization Systems Based on Dominant NGMS

Based on the SPT concept, two dominant male sterility systems have also been developed [56]. Both of these systems utilize the techniques of transcriptional gene silencing through promoter inverted repeats (pIR), to either create male sterility or to restore male fertility [94,95]. In the MS45-pIR system, the *ms* component is the pIR designed to be the promoter of the *TaMS45* gene, which results in dominant male sterility [56]. This sterility can be complemented through the expression of the *MS45* gene with a heterologous promoter from another plant species with an expression profile similar to the *TaMS45*. A second dominant system utilizes the expression of the *DNA (Adenosine-N6-)-Methyltransferase (DAM)* gene in a tapetum-specific manner [56]. The *DAM*-induced male sterility can be restored through the expression of pIRs designed to be the promoter directing the expression of the *DAM* gene. Both the MS45-pIR and the *DAM* hybridization systems can be maintained with a SPT-like TDNA that has the suppression of male sterility component as the complementation piece, along with a seed marker gene and pollen-specific alpha amylase gene. When a *MS*/*MS* plant hemizygous for the TDNA is self-pollinated, all the seeds have a *MS/MS* genotype but segregate approximately 50:50 for with and without TDNA, which can be sorted based on the seed marker. *MS/MS* seeds without TDNA will produce a male sterile female line to be crossed to a male parent for hybrid seed production, while the seeds with TDNA will produce plants in which male sterility is suppressed and thus are male fertile (Figure 3). As opposed to the recessive systems, the male parent needs to be genetically modified for restoration of fertility [56].

## 4. Role of Gene Editing in Developing Next Generation Wheat Hybridization Systems

Gene and genome editing utilizing the emerging tools can speed up crop improvement. Wheat, due its polyploid nature and a large genome size, can especially benefit from these technologies (reviewed in [96]). The utility of gene editing in creating hybridization systems in wheat is immediately evident from the number of triple knockouts of male fertility genes generated for *TaMS26*, *TaMS45*, *TaNP1* and *TaTDF*. For *TaMS45*, the utility of the CRISPR/Cas system was effectively demonstrated through concurrent modifications in all three homeologs for two genotypes, thus allowing simultaneous testing of male sterility in two genetic backgrounds [33]. This demonstrated the utility of gene editing for quickly screening for sources of NGMS that are stable across genotypes. With these editing technologies in mind, researchers are proposing more complex NGMS systems that would not be feasible using the traditional genetics and cytogenetics techniques. The modified 4E-*ms1* and *MFW-PV-OV* are examples of two such systems that will require chromosome engineering, with the *MFW-PV-OV* system requiring gene edits in three genes in addition to large chromosomal deletions [89]. For these proposed hybridization systems, including those utilizing three homeolog edits, trait introgression to different inbreds will be complex and challenging. For introgression of such hybridization systems, one approach would be to leverage the power of genome editing to introduce the hybridization systems de novo to new inbreds, as demonstrated for *TaMS45* [33]. For such a large-scale trait introgression, the efficiency of gene editing will need to be substantially improved. While wheat still lags other model plant species such as Arabidopsis and rice in the utilization of CRISPR-based genome modifications, a couple of recent technological advances point to the maturing of this technology in wheat [97,98]. Next-generation monocot plant transformation systems [99] will also lead to greater efficiency of gene editing in wheat.

## 5. Conclusions

Even though immense progress has been made in understanding the genetic basis of male sterility in wheat, a hybridization system that combines the benefits of lower cost of goods and efficiency has not yet been implemented. NGMS offers potential advantages over CMS with respect to complexity of breeding and hybrid seed production. However, currently all NGMS based systems, particularly those based on non-conditional male sterility, are in the early stages of development, i.e., these are concepts or at proof-of-concept stages. Nevertheless, even at an early stage of development, these concepts look very promising. Some of the complex genetic manipulations that were not possible with traditional genetics or molecular techniques can now be performed through gene editing. Along with a good hybridization system, other important aspects of wheat hybrid seed production that will lower the cost of goods also need to be considered. Technologies and breeding methods to create heterotic pools and enhance cross pollination also need to be developed alongside hybridization systems.

## Figures and Tables

**Table 1:** Mutants and temperature- or photoperiod-variants of the male fertility genes in wheat.

Mutant	Class	Chromosomes	Functional Homeologs	Reference
*Pugsley’s* (*ms1a*)	Recessive	4BS	B	[22]
*Probus* (*ms1b*)	Recessive	4BS	B	[23]
*Cornerstone* (*ms1c*)	Recessive	4BS	B	[24]
*ms1d, e, f*	Recessive	4BS	B	[25]
*Lanhzou* (*ms1g*)	Recessive	4BS	B	[26]
*ms1h*	Recessive	4BS	B	[27]
*Ms2* (*Ta1*)	Dominant	4DS	D	[28]
*Ms3*	Dominant	5AS	A	[29]
*Ms4*	Dominant	4DS	D	[30]
*ms5*	Recessive	3A, 3D	A, D	[31]
*Tams26*	Recessive	4L	A, B, D	[32]
*Tams45*	Recessive	4L	A, B, D	[33]
*Tanp1*	Recessive	1L	A, B, D	[34]
*Tatdf*	Recessive	4L	A, B, D	[35]
*TacalS5*	Recessive	7S	A, B, D	[36]
*Tarpg1*	Recessive	7L	A, B, D	[36]
*Taspps*	Recessive		A, B, D	[37]
4110S	TGMS			[38]
BNY-S (*wtms1*)	Recessive-TGMS	2B	B	[39]
BNS	TGMS			[40]
msBS20T	Recessive-TGMS	2BL	B	[41]
337S	PTGMS	2B, 5B	B	[42]
BS210	PTGMS			[43]
BS366	PTGMS			[44]
C49-S	PTGMS			[45]
XN291S	PTGMS	5B	B	[46]

**Table 2:** Chromosomal location, functional homeologs and alleles, and number of alleles required for complementation of four male fertility genes in wheat.

Gene	Chromosomal Location in C.S.	Number of Functional Homeologs	Number of Functional Alleles for Male Fertility	Number of Alleles Required for Complementation	Reference
*MS1*	4BS	1	1	1	[27]
*MS5*	3A, 3D	2	1	1	[31]
*TaMS26*	4AS, 4BL, 4DL	3	2	2	[32]
*TaMS45*	4AS, 4BL, 4DL	3	1	1	[33]

**Table 3:** Hybridization system concepts for wheat based on non-conditional NGMS.

Strategy	Mutant/Gene	Characteristics	References
**First-Generation Concepts**			
XYZ System	*generic*	Complementation of a *ms* mutation with an alien chromosome; three components for female maintenance XYZ; sorting of *ms/ms* genotype based on vegetative characteristics; 4-line system.	[83]
Modified XYZ System	*Cornerstone (ms1c)*	Wheat or barley isochromosome proposed for complementation of *ms1c*; maintenance of Y line through self-pollination, eliminating the need for X component; A x B like cross required for propagation of Z line; 3-line system.	[84]
**Second-Generation Concepts**			
4E-ms	*Lanzhou (LZ; ms1g)*	Complementation with chromosome 4E from *Agropyron elongatum*. 4E carries the *ms1* homeolog and *Blue Aleurone (BA)* gene; sorting of *ms1/ms1* genotype based on seed phenotype; 2-line system.	[85]
SPT-like System based on recessive sterility	*ms1, ms5, Tams26, Tams45*	Complementation with a TDNA carrying the wild-type copy of the mutant gene; *ms/ms* identification based on the seed marker also present in the TDNA; final product is non-transgenic; can be utilized as a 2-line or 3-line system, whichever is efficient.	[27,31,32,33]
SPT-like System based on dominant sterility	*TaMS45-*pIR	Dominant male sterility; proof of concept transgenic but gene editing can provide a more regulatory-friendly system; manipulation of male lines also needed.	[56]
	*DNA (Adenosine-N6-)-Methyltransferase* *(DAM)*	Dominant male sterility; transgenic; manipulation of male lines also needed.	[56]
SPLIT Gene	*Barnase (B. amyloliquefaciens)*	Based on two components (split) of barnase gene; transgenic; no manipulation of male lines needed; an A x B-like cross needed for maintenance; 3-line system.	[86]
Chemical complementation	*TaSpPS*	Male sterile *Taspps* mutants can produce seed with application of oxo-6-octadecane-pyran-2-olate; registration of chemical for commercial use not determined; 2-line system.	[37]
**Third-Generation Concepts**			
MS45-BA	*Tams45*	Complementation with telosome 4EL from *Thinopyrum ponticum; MS1* and *BA* in tight linkage (same chromosome arm); triple-gene knockouts required for male sterility; 2-line system.	[33,87]
Modified 4E-ms	*ms1*	Complementation with an engineered alien chromosome; *MS1* and *BA* in tight linkage (same chromosome arm); 2-line system.	[87,88]
Updated XYZ	*tip2*	Complementation with barley 7H monosomic addition line; seed sorting based on waxy endosperm/seed phenotype; 3-line system.	[14]
*MFW-PV-OV* Maintainer	*generic*	Requires complex gene editing or transgenic manipulation to combine *MFW*, *PV* and *OV* components in tight linkage; 3-line system.	[89]

## Data Availability

Not applicable.
